# P-1822. Management of Patients with a History of Penicillin Allergy: A Retrospective Observational Study from Saudi Arabia

**DOI:** 10.1093/ofid/ofae631.1985

**Published:** 2025-01-29

**Authors:** Abdullah A Alhifany

**Affiliations:** Umm Al-Qura University, Makkah, Makkah, Saudi Arabia

## Abstract

**Background:**

It has been estimated that 8 to 25% of populations studied globally are labeled penicillin-allergic. However, almost 90% of these patients may not have a true allergy upon work-up, which led prescribers to substitute β-lactams unnecessarily with other broadspectrum antibiotics. Therefore, the aim of this study was to describe how patients with a history of penicillin allergy are treated for infectious diseases, and determine the cost associated with alternative antibiotics.

**Table I.**
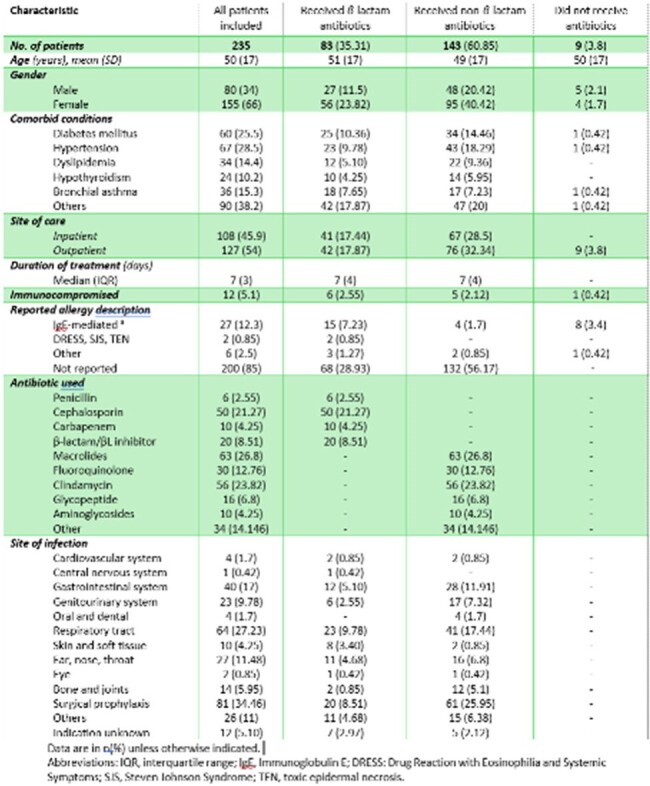
Baseline characteristics of patients with a documented history of penicillin allergy.

**Methods:**

A retrospective observational study conducted in a tertiary-care teaching hospital in Riyadh, Saudi Arabia. Approval was obtained from the Institutional Review Board, and electronic health records (EHRs) of patients were included if they were 18 years old and older, labeled to have penicillin allergy, and were admitted in medical wards or treated as outpatients.

**Results:**

Among the 235 included patients, the type and severity of allergy reactions were not reported in the majority of patients 200 (85%) and were described in only 35 (15%) patients, which 27 (12%) of them had IgE-mediated allergic reactions (angioedema, anaphylaxis, bronchospasm, and urticaria). None of the cases were referred to an immunologist. Most common antibiotic indications included surgical prophylaxis 81 (33%), followed by respiratory 64 (27%) and gastrointestinal infections 40 (17%). For patients with documented penicillin allergy, macrolides were the most used alternative antibiotics followed by clindamycin, cephalosporins, and fluroquinolones in 63 (26%), 56 (24%), 50 (21%), and 30 (12%) patients, respectively.

**Conclusion:**

This study reveals that mislabeling penicillin allergies in Riyadh's tertiary hospital leads to using less effective antibiotics, increasing adverse reactions, infections, and healthcare costs. It emphasizes improving allergy verification to enhance antibiotic use and reduce expenditures.

**Disclosures:**

**All Authors**: No reported disclosures

